# Efficacy of nicotine administration on obsessions and compulsions in OCD: a systematic review

**DOI:** 10.1186/s12991-020-00309-z

**Published:** 2020-09-30

**Authors:** Daria Piacentino, Annalisa Maraone, Valentina Roselli, Isabella Berardelli, Massimo Biondi, Georgios D. Kotzalidis, Massimo Pasquini

**Affiliations:** 1 NESMOS Department (Neurosciences, Mental Health, and Sensory Organs), School of Medicine and Psychology, Sapienza University, Sant’Andrea Hospital, Rome, Italy; 2 Department of Human Neurosciences, Sapienza University, Policlinico Umberto I, Rome, Italy; 3 Clinical Psychoneuroendocrinology and Neuropsychopharmacology Section, National Institute On Drug Abuse Intramural Research Program and National Institute On Alcohol Abuse and Alcoholism Division of Intramural Clinical and Biological Research, National Institutes of Health, Baltimore, MD USA

**Keywords:** Checking behaviour, Cognitive functions, Nicotine, Obsessive–compulsive disorder, Treatment

## Abstract

**Background:**

Preliminary studies have tested nicotine as a novel treatment for OCD patients who respond partially/incompletely or not at all to first and second-line treatment strategies, with the former represented by SSRIs or clomipramine, and the latter by switching to another SSRI, or augmentation with atypical antipsychotics, and/or combination with/switching to cognitive–behavioural therapy. Some studies found nicotine-induced reduction of obsessive thoughts and/or compulsive behaviour in OCD patients. We aimed to evaluate the efficacy of nicotine administration in OCD patients.

**Methods:**

We searched the PubMed, ScienceDirect Scopus, CINHAL, Cochrane, PsycINFO/PsycARTICLES, and EMBASE databases from inception to the present for relevant papers. The ‘Preferred Reporting Items for Systematic Review and Meta-Analyses’ (PRISMA) standards were used. We included all studies focusing on the effects of nicotine administration on OCD patients’ obsessions or compulsions. Studies could be open-label, cross-sectional, randomized controlled trials, case series or case reports.

**Results:**

A total of five studies could be included. Nicotine administration may ameliorate behavioural features and recurrent thoughts of severe, treatment-resistant OCD patients; however, in one study it was not associated with OC symptom improvement or cognitive enhancement across various executive function subdomains.

**Conclusions:**

Although encouraging, the initial positive response from the use of nicotine in OCD needs testing in large controlled studies. This, however, raises ethical issues related to nicotine administration, due to its addiction potential, which were not addressed in the limited literature we examined. As an alternative, novel treatments with drugs able to mimic only the positive effects of nicotine could be implemented.

## Introduction

OCD is a disabling mental health disorder affecting 1.5–3.0% of the general population [81]. Core features of DSM-5 obsessive–compulsive disorder (OCD) include: (a) obsessions, defined as “recurrent and persistent thoughts, urges, or images that are experienced as intrusive and unwanted”, and (b) compulsions, defined as “repetitive behaviours or mental acts that one feels driven to perform in response to an obsession or according to rules that must be applied rigidly” [12]. Obsessions and compulsions (OC) may occupy a significant portion of the day of an OCD patient, with a significant negative impact on his/her quality of life [32]. In fact, obsessions are incredibly difficult to ignore and typically generate great anxiety [51], whereas performing compulsions, such as checking, cleaning, counting, and hoarding, does not necessarily lead to reward or pleasure [39].

The exact aetiology of OCD is partly unknown, yet functional neuroimaging studies point to alteration of the orbitofrontal–basal ganglia circuit [24]. Hyperactivity in the lateral orbitofrontal cortex (OFC) is probably involved in obsessions, while medial OFC hypoactivation could be associated with extinction recall and dysfunctional inhibitory control [66]. The amygdala and the dorsal anterior cingulated cortex are also involved, respectively, in error monitoring and in exaggerated fear responses. More recent neuroimaging and psychophysiological data suggest that compulsions might represent the core of OCD, while obsessions might arise as a result of compulsions, leading to the hypothesis that obsessions could represent a subsequent mentalization process [42, 43]. As for the pathophysiology of OC symptoms, the prevailing view involves a dysfunction of the serotonergic, dopaminergic, and glutamatergic pathways [13, 49, 63].

Several guidelines and algorithms have been developed through the years to tackle the issue of the treatment of this hard to manage psychiatric disorder [11, 18, 35, 72], that may impair the patient’s everyday life and social performance [3, 76, 91]. However, the validity of treatment guidelines is commonly accepted to last about two years after their publication, so most of them are obsolete. Besides this, there has been a reclassification of OCD, that was deprived of hoarding disorder [77] and moved from the anxiety disorders to a spectrum of its own in the DSM-5 [12]. This has important treatment implications, as hoarding cannot be currently treated with psychopharmacological means, and the use of anxiolytic drugs, which proved to be ineffective in OCD, has been set off. The established first-line drug treatment for OCD includes antidepressants increasing serotonin in the synaptic cleft [54], such as selective serotonin reuptake inhibitors (SSRIs), mainly due to their more benign side effect profile. Evidence is also strong for clomipramine [31] and until the 1990s it was considered to be the gold standard [33, 34, 98]. As there is no evidence for the efficacy of other tricyclic antidepressants in OCD, the efficacy of clomipramine has been attributed to its effect on serotonin (5-HT) [79] and dopamine (DA) [15]. Unfortunately, about 40% of OCD patients do not achieve an acceptable remission of OC symptoms in response to SSRIs or clomipramine, among those who do respond, the response is often partial or incomplete [5, 6]. Even when switching to another SSRI, the percentage of non-responders remains high, about 30% [55]. Hence, augmentation with atypical antipsychotics is an established second-line drug treatment strategy, alternatives include combination with or switching to cognitive–behavioural therapy (CBT [5, 6].

Despite progress in the understanding of the molecular underpinnings of OCD, its treatment status is unsatisfactory. About 40–60% of patients are resistant to first-line treatment [62], but there is no general consensus as to what is treatment-resistant or -refractory OCD, although some investigators still retain the criterion of a lack of response (i.e. less than 25 or 35% drop in Y-BOCS scores from baseline, according to individual studies) after at least two SSRI and/or clomipramine trials [90]. Given the above considerations, there is need for alternative OCD treatments.

Rat models of OCD have facilitated research for novel treatments, such as nicotine [92–94, 99]. Tizabi et al. [99] observed the onset of compulsive checking behaviour in rats treated with the D_2_–D_3_ dopamine receptor agonist, quinpirole. This paradigm was suggested as an animal model of OCD. Nicotine was shown to attenuate some symptoms of compulsive checking in these previously sensitized rats. The same did not occur in non-sensitized rats. The role of dopamine in the triggering of OCD symptoms is long recognized [47, 57]. Human data show increased dopaminergic activity in the striatum in OCD, as evinced by D_1_ receptor down-regulation [74]. More recently, it became apparent that nicotine controls dopaminergic activity in the striatum. The chronic stimulation of α_6_β_2_ and/or α_4_β_2_ nicotinic receptors slows down dopaminergic firing in the striatum [38]. This prompted our interest to study the effects of nicotine agonists in OCD.

Preliminary studies of nicotine administration in OCD patients showed a reduction of compulsive behaviour [30, 59, 75, 82] and, in some cases, of obsessive thoughts [30, 59, 75]. Since these preliminary studies pointed to the possible utility of nicotine agonists, we decided to perform a systematic review of nicotine or nicotinic agonists as a treatment for OCD.

The aim of this systematic review is to summarize data assessing the efficacy of nicotine or nicotinic agonist administration in OCD. Research data are currently few and conflicting.

## Methods

We followed the ‘*P*referred *R*eporting *I*tems for *S*ystematic Review and *M*eta-*A*nalyses’ (PRISMA) indications in study identification and selection [69]. We searched the PubMed, ScienceDirect Scopus, CINHAL, Cochrane Library, PsycINFO/PsycARTICLES, Web of Science/Clarivate, and EMBASE databases from their inception to May 18–19, 2020, to identify peer-reviewed studies examining the effects of nicotine administration in OCD patients. The PubMed search was (OCD [title/abstract] OR obsess* [title/abstract] OR compuls* [title/abstract]) AND nicotine [title/abstract] and was adapted according to each database’s needs. The reference lists of located papers were examined and cross-referenced for further relevant literature. We included studies with both therapeutic intent as well as those with other main outcomes, but which investigated OCD symptoms as related to nicotine intake. We included studies independently from administration route. We excluded studies in which OC were part of other OCD spectrum disorders like hoarding disorder, excoriation (skin-picking) disorder, substance/medication-induced OC and related disorder, OC and related disorder due to another medical condition, and trichotillomania (hair-pulling disorder), or tic disorders, or Tourette syndrome, or OC personality disorder. We also excluded animal studies, postmortem or in vitro studies or studies with nonclinical outcomes. Inclusion criteria are shown in Table 1.

**Table 1:** Inclusion criteria

Study design	Open-label, cross-sectional, randomized controlled trials, case series, case reports
OCD diagnosis	OCD diagnosis according to DSM-III, DSM III-R, DSM-IV, DSM-IV-TR, or DSM-5^a^
Assessment methods for clinical improvement	Self-, physician- or computer-administered questionnaires, semi-structured interviews
Outcomes	Reduction of obsessions and/or compulsions
Languages of publication	Any language
Country	Any country
Year of publication	Any year
Population	OCD patients
Gender	Male and female
Age range	18–65 years

^a^[7–10, 12]

## Results

**Fig. 1:**
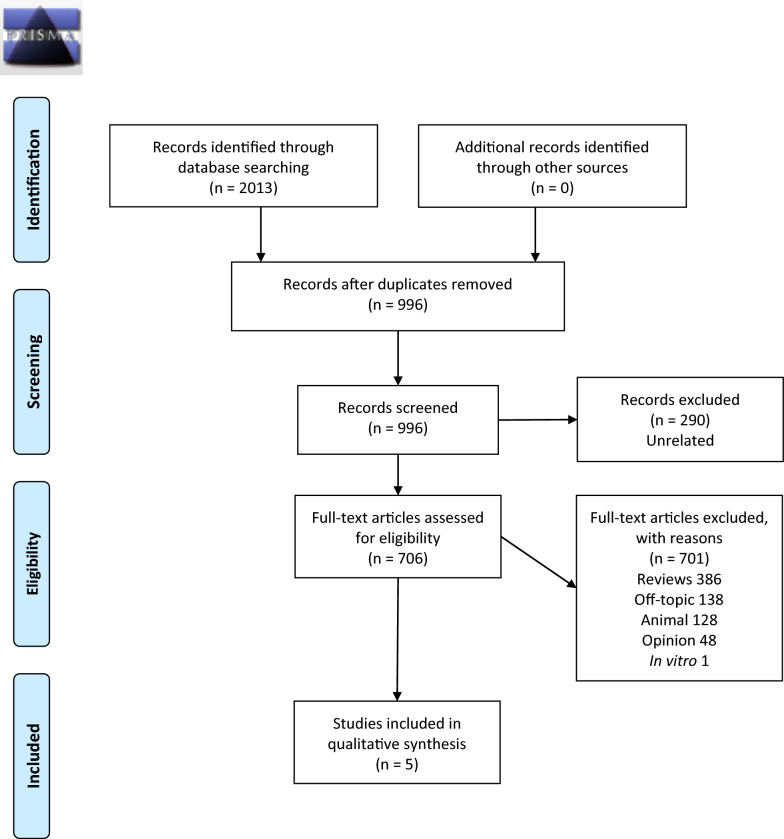
PRISMA flowchart for inclusion of studies in the systematic review with reasons for exclusion

In PubMed, on May 18, 2020, the above search produced 232 records; on Embase, on May 19, 2020, ('nicotine'/exp OR nicotine) AND (obsessi* OR compulsi*) produced 557 records; on Scopus, TITLE-ABS-KEY ( ( obsessi*) OR ( compulsi*)) TITLE-ABS-KEY ( nicotine) on May 18, 2020 yielded 520 records, Title-Abstract-Keyword nicotine AND (obsessi* OR compulsi*) on Cochrane Library on May 18, 2020 produced 30 records; nicotine AND (obsessi* OR compulsi*) on CINAHL produced on May 18, 2020 yielded 57 records, while the same search on PsycLIT (PsycINFO/PsycARTICLES) on the same date produced 311 records, which were reduced to 306 after elimination of duplicates; finally, nicotine AND (obsessi* OR compulsi*) on Web of Science produced on May 18, 2020 311 records. Further studies were sought on ClinicalTrials.gov on May 19, 2020, but none was found. The reference lists of all located articles were further reviewed to detect still unidentified literature, but produced no new articles. The selection procedure and reasons for exclusion of studies are shown in Fig. 1.

A total of 5 studies focused on the effects of nicotine in OCD. They involved a sample size that ranged from 1 (i.e. case reports) to 40 individuals (i.e. cross-sectional study), with a mean of 10.6 and a median of 5.0, indicating skewness towards smaller samples. There were four studies with therapeutic intent and one with another outcome (differences between smokers and nonsmokers in cognitive performane), but which provided data on OCD symptoms [28]. The following data were extracted from included studies: study source (i.e. first author’s name and year of publication), country of origin; study design; sample size; method and type of nicotine administration; assessment methods; key findings, with a focus on the effects of nicotine on OC symptoms. Extracted data are listed in Table 2.

**Table 2:** Data extracted from the selected studies (*N* = 5)

First author/s (year of publication)	Country	Study design (number of participants)	Method and method of nicotine administration	Assessment methods	Key findings	
*With treatment as outcome*						
Carlsson and Carlsson [30]	Sweden	Case report (*N* = 1)	Monotherapy; 2 mg chewing gums × 8 weeks, then > 1 year with doses escalated by patient	Y-BOCS		1 DSM-IV OCD outpatient. ↓ Y-BOCS total score (45.45%) and obsession and compulsion subscale scores
Salín-Pascual and Basañez-Villa [82]	Mexico	Randomized controlled (*N* = 11)	Monotherapy; 17.5 mg transdermal patches × 5 consecutive days	Y-BOCS, BAI, BDI		6 DSM-IV OCD female and 5 DSM-IV OCD male outpatients. ↓ Y-BOCS total score (36.20%) and compulsion subscale scores. ↓ BAI scores. No changes in BDI scores
Lundberg et al. [59]	Sweden	Case series (*N* = 5)	Monotherapy or add-on therapy; 17.5 mg transdermal patches × 3 weeks, then 2 mg chewing gums × 5 weeks	Y-BOCS, NIMH-GOCS, BAI, BDI, GAF		5 DSM-IV OCD outpatients. ↓ Y-BOCS total score (26.63%) and obsession and compulsion subscale scores, as well as ↓ NIMH-OCS scores, in 3/5 (60%) patients. ↓ BAI and BDI scores. ↑ GAF scores (7.84%) after 15 weeks
Pasquini et al. [75]	Italy	Case report (*N* = 1)	Add-on therapy; 4 mg chewing gums × 4 months	Y-BOCS, CGIs		1 DSM-IV-TR OCD outpatient. ↓ Y-BOCS total score (39.50%) and obsession and compulsion subscale scores. ↓ CGIs scores (= 2)
*Evaluation of cognition, but providing data on OCD symptoms*						
Caldirola et al. [28]	Italy	Cross-sectional (*N* = 40) comparison of smokers (*N* = 20) *vs*. nonsmokers (*N* = 20)	Usual smoking habits (cigarettes or no nicotine intake); all 40 patients were on SSRIs, tricyclic antidepressants, and/or antipsychotic drugs	Y-BOCS, CANTAB		40 DSM-IV-TR OCD inpatients. No difference in Y-BOCS and CANTAB scores between smokers and nonsmokers. The longer-term smokers had more cognitive impairment

*BAI* Beck Anxiety Inventory, *BDI* Beck Depression Inventory, *CANTAB* Cambridge Automated Neuropsychological Test Battery, *CGIs* Clinical Global Impressions Scale-severity, *GAF* Global Assessment of Functioning, *NIMH-GOCS* National Institute of Mental Health Global Obsessive–Compulsive Scale, *Y-BOCS* Yale-Brown Obsessive–Compulsive Scale

All studies employed the Yale-Brown Obsessive–Compulsive Scale (Y-BOCS) [45, 46], the gold standard physician-administered, semi-structured interview for assessing OCD symptom type and severity. In addition to the Y-BOCS, Lundberg et al. [59] used the National Institute of Mental Health Global Obsessive–Compulsive Scale (NIMH-GOCS) [53], a physician-administered questionnaire measuring OCD severity, and Caldirola et al. [28] used the Cambridge Automated Neuropsychological Test Battery (CANTAB) [80], a computer-administered combination of 25 neuropsychological tests that is largely used in OCD [1].

Of the four therapeutic studies, all three case reports/series used chewing gums [30, 59, 75] and the only randomized control trial [82] used 17.5 mg transdermal patches, as did the case series, which entailed a 2-mg gum extension [59]. The latter was a monotherapy or add-on, while one case report and the randomized study were monotherapy alone [30, 82] and another case report an add-on [75]. Two studies used 2 mg chewing gums [30, 59] and one a 4-mg dose of chewing gum added on 200 mg clomipramine and 1000 mg valproate in a treatment-resistant patient [75]. Treatment durations were variable, from 5 days in the randomized study to a mean of 8 weeks to years (Table 2). The non-therapeutic study was cross-sectional.

Carlsson and Carlsson [30] and Pasquini et al. [75], in their case reports of two OCD patients with a 13- and 16-year history of the disorder, respectively, who were refractory to all drug classes (i.e. SSRIs, tricyclic antidepressants, antipsychotics, mood stabilizers, and benzodiazepines), found marked symptom improvement after nicotine administration. Carlsson and Carlsson [30] observed a decrease in Y-BOCS total, obsession, and compulsion scores after 8 weeks of nicotine treatment with 4 gums/day (2 mg gums) and discontinuation of all other drugs in a 37-year-old woman with OCD. The patient further improved after 10 months, after having autonomously increased nicotine gum consumption from 4 to 10 gums/day. In fact, self-administered dosing allowed her to increase workload and effectively tolerate insomnia-elicited stress, which had previously been a powerful inducer of obsessions. She also reported a general reduction of the traffic of thoughts and images.

Pasquini et al. [75] administered 1 gum/day (4 mg gums) to a 31-year-old man, in addition to clomipramine 200 mg and valproic acid 1 g, for 4 weeks and obtained satisfactory results, confirmed by both the patient and his relatives. Patient’s Y-BOCS scores dropped satisfactorily from baseline to the 4th week on total, obsession, and compulsion scales. The patient improved also on the Clinical Global of Impressions-severity scale (CGIs; [48]); at week 4 he had a score of 2 (borderline mentally ill). The patient obtained benefit and developed no side effects for another three months, allowing to reduce doses of other drugs.

Salín-Pascual and Basañez-Villa [82] assessed 11 nonsmokers with OCD who were double-blind randomized to 17.5 mg transdermal nicotine patches or placebo for 5 days. Patients were asked to rate their obsessions and compulsions, anxiety, and depression. Nicotine treatment reduced Y-BOCS total and compulsion scores, but not obsession scores, compared to placebo patches. Nicotine was associated to a decrease of Beck Anxiety Inventory (BAI) [20] scores, but not Beck Depression Inventory (BDI) [19] scores, compared to placebo.

Lundberg et al. [59], after observing the striking effects of nicotine self-medication in the OCD patient described by Carlsson and Carlsson [30], continued to treat this patient and another four, with 17.5 mg transdermal patches for 3 weeks, followed by 1–10 gums/day (2 mg gums) for the remaining 5 weeks. The high variability in the number of gums was a result of the patients increasing consumption over time. In the five patients, mean consumption was of 1.7, 1.1, 2.8, 1.6, and 2.0 gums/day, respectively; OCD duration was 5, 8, 10, 13, and 17 years, respectively. Except for one patient, all others had previously received SSRIs and/or tricyclic antidepressants. All patients, prior to entering the study, had completed a CBT programme. After 8 weeks, nicotine was discontinued for 8 weeks to avoid nicotine dependence. After the first 8 weeks, four patients showed a reduction of Y-BOCS total, compulsion, and obsession scores. They also showed a reduction of the NIMH-GOCS total score. Furthermore, BAI and BDI scores decreased, indicating improvement in anxiety and mood, while Global Assessment of Functioning (GAF) [89] scores increased, indicating better functioning. Patients attributed this to the less intense traffic of disagreeable thoughts and images and to better coping with stress. A role for CBT in supporting the effects of nicotine in the brain could be hypothesized. Three of the five patients were satisfied with their nicotine treatment and two were not. Of the latter, one had to quit treatment due to OC symptom worsening. Mild side effects were reported by three patients and consisted in nausea and shortness of breath.

Caldirola et al. [28] performed a cross-sectional study, comparing, 1–3 days after hospitalization, 20 OCD nonsmoker with 20 OCD smoker inpatients (mean smoking career 25.9 years; mean number of cigarettes 24 h before assessment in smokers, 12.5; mean number of cigarettes on the day of the assessment, 3.5, time not provided) on the Y-BOCS and on cognitive measures. All 40 were receiving SSRIs, tricyclic antidepressants, and/or antipsychotic treatment. No significant difference in terms of OCD severity or cognitive performance was found between smokers and nonsmokers, as shown through the Y-BOCS and CANTAB scores. The authors assessed executive function subdomains which were previously found to be impaired in OCD [1, 85, 88]. Smoking duration significantly correlated with an increased number of spatial working memory errors. Their findings suggest a lack of beneficial cognitive effects of nicotine, but the presence of long-term nicotine-associated cognitive impairment. However, this study cannot be taken as a study of nicotine administration in OCD patients with the intent to treat their OC symptoms, but rather to observe differences in OC symptoms (and cognition) in patients who were on tobacco smoking since some time and in those who abstained. They observed no differences.

## Discussion

We reviewed evidence for nicotine treatment of OCD. The literature on this subject, in spite of the existence of sound preclinical evidence [92–94, 99], is surprisingly scanty. Despite few studies, the methods of nicotine administration and dosing were multiple and inconsistent. Evaluated were the efficacy of nicotine, administered as cigarettes, chewing gums, or transdermal patches, in severe, treatment-resistant OCD. Compulsions [30, 59, 75, 82] and, less frequently, obsessions [30, 59, 75] decreased. One of the included studies [28] failed to find any effects of nicotine on OC symptoms and cognitive performance; smoking did not enhance visuospatial working memory, planning, or set-shifting abilities in OCD patients. However, this study was not designed to assess the effects of nicotine administration on OC symptoms, but rather to assess the effect of long-term smoking habit on OC symptoms and cognition. Since it was a cross-sectional study, it cannot be taken seriously as a test of the effects of nicotine in OCD. The finding of reduced executive performance in OCD patients with longer-term nicotine use contrasts with the findings of the use of nicotine in other mentally ill populations, like schizophrenia, major depressive disorder, bipolar disorder, and attention-deficit hyperactivity disorder (ADHD). In these populations, smokers performed better on executive tasks [27, 67, 70, 103, 104]. Caldirola et al. [28] speculated that since nicotine enhances cortical activity by promoting the release of ACh, 5-HT, DA, NA, GABA, and Glu [102], the nicotine-induced activation of an already hyperactive system of OCD patients may not ameliorate cognitive performance and render these patients less likely to engage in smoking with respect to other patient populations. Prevalence rates of cigarette smoking are typically higher among individuals with psychiatric disorders (36–64%) than among the general population (19–24%) [2]. The ‘self-medication hypothesis’ for schizophrenia and mood disorders assumes that smoking ameliorates clinical symptoms and attentional deficits [102, 103]. According to the nicotine receptor hypothesis, ADHD is a risk factor for earlier onset and higher rates of cigarette smoking, basing on the conjecture that nicotinic receptors modulate dopaminergic activity and that dopaminergic dysregulation may be involved in the underlying pathophysiology of ADHD [67]. In contrast, OCD is associated with lower smoking prevalence rates, around 13–14% [21, 70], albeit with (a) geographic differences [36], (b) gender distinctions, with OCD females more prone to smoke than their male counterparts [36], (c) co-occurrence of other disorders, such as tic disorders, Tourette syndrome, and major depressive disorder [2, 36], (d) specific symptom dimensions, with lower smoking ratios in the ‘washing’ group, intermediate ones in the ‘taboo thoughts’ group, and higher ones in the ‘symmetry–counting–repeating–ordering’ group [96], and (e) personality traits, with nonsmokers characterized by low self-confidence, low impulsiveness, and pronounced OC personality disorder traits [22].

It should be added that nicotine has been tested with mixed results for the treatment of behavioural dysfunction (i.e. tics, compulsions) and attentional impairment in patients with Tourette syndrome [52, 83, 86, 97]. The latter often overlaps with OCD in ways that suggest a tantalizing relatedness, although the formal barriers of DSM-5 differential diagnosis cannot be ignored. Existing studies overall suggest that additional work is required to better characterize the behavioural and cognitive effects of nicotine in treatment-refractory Tourette syndrome [52]. A better understanding of the pathophysiology of the disorder with regard to cholinergic, glutamatergic, and histaminergic neurotransmission is needed [50].

### Rationale of nicotine administration: neurotransmitter pathways in OCD

While there is consensus on the importance of DA [37, 49], and glutamate (Glu) pathways [25, 63] in the pathophysiology of OCD, the role of the cholinergic system is less clear. Significantly higher pseudocholinesterase levels were found in OCD patients compared with age- and gender-matched healthy volunteers [4]. Cholinergic supersensitivity in OCD patients has been advanced to explain elevated growth hormone responses to the ACh esterase inhibitor pyridostigmine [58]. Studies using magnetic resonance spectroscopy showed significantly higher levels of choline, the precursor of acetylcholine (ACh), in the thalamus of OCD patients compared with patients with major depressive disorder and with the general population [87]. In addition, the first-line treatment, clomipramine, inhibits most muscarinic receptors throughout the body, but those that are considered to be important for OCD are mainly located in the OFC [29]. This mechanism does without nicotine receptors, but Carlsson [29] supports a role for such receptors in OCD, in that both clomipramine and nicotine reduce ACh release in the striatum. In the same line, a recent study on patients with treatment-resistant OCD who had shown partial or no response to multiple SSRIs, observed that add-on of the reversible acetylcholinesterase inhibitor donepezil, which upregulates nicotinic cholinergic receptors (nAChRs), was well-tolerated and produced symptom improvement [23]. The positive response to donepezil suggests a central role for nAChRs.

### Rationale of nicotine administration: nicotine’s mechanism of action

Nicotine’s mechanism on OC symptoms is incompletely understood. Nicotine selectively activates different nAChR subtypes. By interacting with some presynaptic nAChRs, nicotine determines the release of several neurotransmitters, such as ACh, 5-HT, DA, norepinephrine (NA), γ-aminobutyric acid (GABA), and Glu [102]. The role of nAChRs in behavioural tasks in which all the above transmitters are involved [71, 78] prompted to consider these receptors in some psychiatric disorders, such as Tourette syndrome, schizophrenia, and autism spectrum disorders [64]. The interest for the nicotine receptor stimulation in OCD resides in the fact that the high sensitivity α4β2-bearing nAChR neurons are distributed along neural pathways involved with OCD and that such receptors appear to stimulate striatal DA release [68]. Furthermore, compulsive behaviour in a mouse paradigm was shown to be affected by both α_4_β_2_ and α_7_ nAChRs in the islands of Calleja, an important structure for reward, with the latter reducing grooming behaviour upon stimulation, while the former exhibited a more complex action [65]. Nicotine promotes glutamatergic transmission and induces long-term potentiation of excitatory hippocampal [41] and ventral tegmental area input [60], and stabilizes glutamatergic activity of the OFC–cingulate–striatal–thalamic loop [14, 44, 61, 101]. In OCD, hyperactivation is higher in the cholinergic rather than in the glutamatergic system [29]. Nicotine enhances GABAergic transmission transiently, this is followed by a persistent depression of these inhibitory neurons, due to nAChR desensitization. Simultaneously, nicotine enhances glutamatergic transmission through nAChRs on Glu neurons, which desensitize less and later than those on GABA neurons. The net effect is a shift toward excitation, hence with a reduction of inhibitory input [61]. In the prefrontal cortex and hippocampus, chronic nicotine exposure induces 5-HT transporter upregulation [16], presumably through stimulation of α7 nAChRs [17]. ACh in the hippocampus has a dual effect, i.e. inhibition of 5-HT release through M1 muscarinic receptors and enhancement through nAChRs, but while the nicotinic antagonist mecamylamine antagonizes this effect, at high concentrations it has a 5-HT releasing effect [56]. Reduced striatal ACh release and decreased OFC hyperglutamatergia could be potential mechanisms through which nicotine acts on compulsions [29]. Moreover, OFC hyperactivation was found with positron emission tomography during nicotine craving [26]. An alternative explanation of the effects of nicotine in OCD takes into account its effect on memory functions. Some authors found deficits in nonverbal memory and memory for actions to be common among OCD patients [40, 73, 84, 95, 105], especially their own recall, due to pathological doubt (‘la folie de doute’) and low confidence in memory [100], suggesting alterations in cholinergic transmission. Hence, the effects of nicotine on compulsions could be attributed to an increase of memory for actions and to reduced OFC activation, which could be associated to lack of memory inputs [75].

With this data at hand, we cannot recommend the use of nicotine either alone or as add-on in the treatment of OCD. Several questions remain unanswered. For how long should we treat patients before tangible symptom improvement becomes apparent? In the double-blind study [82], the positive effects were observed just after 5 days of treatment, but in the case reports/series, the time to response was variable. Is there any worry about passing the patient from one compulsion to another, i.e. substance use disorder? In the seminal case report [30], the patient, after a period of well-being, passed to dose escalation. We believe the response could be drug design of nicotine agonists that show a better effect size at testing, being free from “abuse” potential.

The strength of this systematic review relies on the multiple database search and the adhesion to the *PRISMA* statement. Its main limitations are: (1) the small number of includible studies, and the small sample investigated; (2) the extreme methodological variability, with heterogeneity in both duration of treatment and treatment modalities, and (3) the lack of sufficient randomized controlled trials and open-label studies, which limit the possible generalization of our findings. Additionally, these findings should be viewed with caution, since nicotine administration was conducted through different modalities in the included studies, which did not report on important measures like severity, comorbidities, and nicotine or its metabolite blood/saliva levels.

Taken together, our review does not allow us to recommend nicotine as a third-line add-on treatment to overcome treatment resistance in OCD nor as monotherapy. Encouraging patients to smoke may be ruled out as it may promote substance use disorder, but other routes may be trialled. However, controlled studies are needed to better assess this issue. It should be underlined that there was just one therapeutic study (of low quality) that was a randomized controlled trial and used transdermal patches [82]. Future studies should standardize their interventions, set inclusion/exclusion criteria with cut-offs based on the Y-BOCS and response/remission criteria based on the same scale, and set specific timepoints for assessment (we feel that 8 weeks is sufficient for acute studies, a 3-month follow-up is better for medium term studies). These studies should use clear and commonly accepted drug-resistance and treatment-resistance criteria. We here presumed treatment resistance in individual patients on the basis of the multiple treatments received and the long-standing illness duration, but when having to include patients in a randomized clinical trial, matters change.

## Conclusions

Nicotine may ameliorate OC symptoms in severe, treatment-refractory OCD patients. Although encouraging, these initial positive effects should be tested in large controlled studies. This, however, raises ethical issues related to nicotine administration, due to its addictive potential, which were not addressed in the limited literature we examined. As an alternative, novel treatments with drugs able to mimic only the positive effects of nicotine could be implemented.

## Data Availability

N/A
